# Aseptic and Antiseptic Dentistry

**Published:** 1890-06

**Authors:** H. M. Clifford

**Affiliations:** Boston, Mass.


ARTICLE IV.

### ASEPTIC AND ANTISEPTIC DENTISTRY.

BY H. M. CLIFFORD, D. M. D., BOSTON, MASS.

[Read before the Harvard Odontological Society, March 27,1890.

Aseptic and antiseptic surgery, special and general, is interesting, if not of great practical importance to the general dental practitioner. To the more careful and pains-taking dentist, the subject has considerable import.

My attention to this subject was awakened more than ever by the very interesting and instructive line of experiments conducted by Dr. H. L. Burrell and assistant, G. R. Tucker, of this city, and given in a paper before the Massachusetts Medical Society. The deductions, made from most excellent investigations, struck me as those which should more directly reach the ears of the dental profession; and as I have seen no record of similar experiments in any dental journal, I resolved to make this subject the theme of my paper. Not having time to enter into experiments of this nature myself, you will accept that which is especially interesting or applicable to our specialty.

Our knowledge of that science which is so successfully aiding the present operations of eminent surgeons, is not extensive; and we shall profit in noting the work of the more advanced scientists in our own ranks—Black, Miller, Sudduth, and many others.

The science of bacteriology furnishes great aid to practical surgery at the present time. The full benefit of this science will not be realized until schools of medicine (and I hope dentistry will be continued as one of its special branches) educate their students in this direction, and surgeons become more familiar with both the principles and details of the work.

Aseptic healing of wounds was long ago heard of.

Fortune favored operations by excluding or destroying the germs, no matter what precautions were taken. Detail after detail has arisen, had its day, and died out; changes will continue, but beneath this rests the foundation which moveth not; the principles of asepticism and antisepticism. That sources of contamination exist in every detail of operative surgery is shown by the experiments I quote below.

I will first mention the result of experiments made on the toilet of the surgeon. The most scrupulous care should be exercised in providing for cleanliness of the hands. Its applications to us as dentists is not so absolutely necessary, but will simply show how careful a person may be and yet be loaded to the finger tips with germs. Note the following experiments:

A man's left hand was selected, the nails cut and cleansed, the hand vigorously scrubbed with soap and water, washed with ether and immersed in one to one thousand (1-1000th) solution of corrosive sublimate. It was kept immersed for one hour, removed from the sublimate bath, washed with sterilized water, and a layer of gauze placed on the front and back of the hand. This was covered with sterilized nutrient gelatine, and over this was applied an antiseptic dressing. The man's right hand was then cleansed in the way a surgeon often does before an operation—that is, by cleansing the nails of all visible dirt; scrubbing the hand with soap and water, and immersing it for a moment in a one to one thousand solution of corrosive sublimate. It was rinsed in sterilized water and treated similarly to the left hand.

A half hour later, these dressings were removed and cultures made. The left hand, the one with which great care was taken, was absolutely sterile. The gelatine of the right hand, the one in which the customary precautions were taken, showed colonies of bacteria; more from the back of the hand than from the front.

The result is striking, and demonstrates that the ordinary way of cleansing the hands is ineffectual. Nail brushes were examined and found to be swarming with bacteria.

A noticeable fact in connection with the above experiment is, that corrosive sublimate, momentarily applied, is not absolutely germicidal. It requires a longer time to act in the strength of one to one thousand than has heretofore been expected.

As corrosive sublimate is destructive to instruments, it was hoped hydronapthol, which is perfectly harmless, would successfully sterilixe them, but it was found to be unreliable in the experiment of a bulldog forcep immersed six hours in an alcoholic solution (1 to 500), complete liquefication from the germs being the result. In a knife treated the same way, better results were realized, the knife being found perfectly sterile.

Experiments by heat were made, and found to be very efficacious.

Sterilization by interrupted heat, leaving interruption for development of spores, is a most certain method.

Boiling, unless interrupted, is not an absolute means of sterilization.

Continuous heat is quite effectual.

A few instruments, after being sterilized, fell upon the floor, and were again examined, and found to be covered with germs; showing the atmosphere of the room, as well as the floor, to be full of germs.

An experiment on instruments by using carbolic acid one to forty, submerged for one hour, showed bacteriologically to be little better than those not treated at all.

Experiments with steam were not satisfactory.

Baking for a few hours gave certain results.

Great care should be exercised that no germs are retained within a wound. Operative wounds, if properly treated, are aseptic, as in the immediate destruction of a pulp. Accidental wounds are more or less septic, as in dead pulps of long standing.

Antiseptic dressings, as carbolized or boracic acid and iodoform cottons, are rarely sterile.

Gauze, saturated with a ten per cent. solution of glycerine, dried and baked, makes a most perfect aseptic dressing; if this same dressing is charged with corrosive sublimate, it makes an admirable antiseptic absorbent.

That germs come from the circulatory system, seems to be proven by an experiment on dressings removed from a wound which had healed without pus; the outside layers having no germs, while the layers immediately in contact with the skin, contained considerable numbers. The germs not coming from outside sources, must come from within.

After considering these experiments, it seems surprising that we as dentists have anything like success in treating diseased mouths and teeth. The knowledge gained from these experiments, if carefully and intelligently applied to dental operations, must have its good effect. Nearly all the observations made by Dr. Burrell should be applied by dentists in treating diseased mouths, exposed and inflamed pulps, and in filling and treating root canals. That a single operation heretofore performed by dentists was bacteriologically prophylactic, I sincerely doubt.

Antiseptic precautions should be more closely observed. I have been in the habit, of late, of treating all foul teeth by the most thorough germicidal applications, namely, corrosive sublimate, 1 to 500, heat, etc., applied with the rubber dam in position.

As the dose of bichloride of mercury, internally administered, ranges from 1-30th to 1-10th of a grain, and being immediately absorbed and giving no poisonous results, it doesn't seem possible that a quantity necessary to a foul pulp or abscessed tooth should give any fear of ill effects. I do not recommend it for anything but foul pulp canals, and then to be used with a knowledge of the significance of the drug. When not using the bichloride as above, I syringe the root canals with a solution of corrosive sublimate, 1 to 1,000 dry, pump up hydrogen peroxide, wash with ether—when the odor is not objectionable—dry with hot air, then apply the root canal dryer, which I consider, at present, the best means of applying heat directly to the roots, keep that in the canals until the roots are practically dry, then I apply a pledget of cotton, moistened with the oil of peppermint or deliquesced carbolic acid and seal up; being careful not to plug the root too tightly. I repeat this as often as I consider necessary, which is rarely oftener than twice, perhaps three times, intervals between such treatment being about a week or ten days.

When the roots appear to be restored to a healthful condition, I proceed to fill with oxychloride of zinc, which, if carefully and intelligently applied, makes one of the best of root fillings. A root thoroughly sterilized will not, when filled with a good filling (e. g., oxychloride) be troubled with an abscess. Oxychloride in a partially sterilized root, when irritating the parts beyond, by its affinity for moisture, will resolve itself without giving further trouble. If pushed through the apex, it will undoubtedly give trouble from its escharotic action, with its consequent evil effects.

It has been said by Virchow and others that the human organism may be considered as a vast battle-field, in which there is a constant conflict between the component cells and micro organisms. Disease or inhibition of the cells gives to the micro-organisms an opportunity to gain the ascendency; destruction of the tissue, putrefaction and pus formation are the results. Miller's experiments (*Independent Practitioner*, 1884 and 1885) prove oxychloride, when first mixed, to be a powerful antiseptic. If it does its work when first introduced into a root canal, and properly excluded from the action of the saliva and mucous of the mouth, it comes as near being a perfect antiseptic root filling as any material we have at our command. The objection of disintegration cannot apply to this material, when used as a root filling. Under a securely tight capping, like a copper amalgam, the only way in which disintegration could take place would be through the apex by the circulatory system, which, to my mind, is very doubtful. In teeth with large apical formina, I would not advise the use of oxychloride as a root filling. I never attempt to fill roots with this preparation when patients are not in the best of health, preferring to use a material I could remove very easily and quickly, if the case called for it.

I never had occasion to regret the use of oxychloride as a root filling but once; and I must confess it was a sad mistake, or accident. For the benefit of those who, I hope, have never experienced the same result, I will relate this operation.

I was treating for neuralgia. Everything was conditioned, with the exception of a left superior lateral, which had been filled on both the mesial and distal aspects with large gold fillings, done by a reputably good operator, and gave every indication of being a perfectly healthy tooth, good color, etc. Upon further examination, however, I found on the gum, a point which looked as if it might at one time have been a fistula. The patient also stated there had been one. The fistula having healed, I was loath to probe it, so I proceeded to enter the root canals. The root canals proved to be free from any foul odor. I applied dressings, sealed, and gave the patient another appointment. At the next appointment, after cleansing and using the customary precautions, I proceeded to fill with my oxychloride. I noticed it was taking more material than usual, became curious, investigated, and found the parts in the neighborhood of the closed fistula, which was heretofore apparently normal, betraying a disturbed condition, probed and found my oxychloride pushing toward the opening. I predicted trouble, used my syringe, applied oil of cajuput and tannic acid, hoping to prevent or counteract the escharotic action of the oxychloride, and dismissed the patient, telling her to call on the morrow. She did so, after passing a sleepless night. The parts were very much inflamed and very sore. In a few days the inflammation subsided, and the part corresponding to the fistula sloughed, which, upon being removed, exposed the condition of the root. From its chronic condition, the labial part of the root, as well as the alveolus on the same side, had been completely dissolved or absorbed, leaving sharp corners and rough edges to cause irritation. I was at a loss to know what to do, and sought the counsel and advise of our ex-president, Dr. Briggs. Suffice it to say, that after following his advice, the patient's neuralgia was cured.

It was also necessary to bring into requisition the services of Dr. Stoddard in baking a piece of gum porcelain which perfectly matched the natural gum; and now a healthful looking tooth is presented, with all traces of neuralgia removed.

Since that time, I have occasionally adopted other antiseptic fillings for root canals. One which I sometimes use, and consider a very fair root filling, is a cement made of tannic acid and a strong solution of gum benzoin in alcohol, and evaporated to a thick consistency. It makes a resinous cement, which forms quite a solid body. I have also applied it in deep-seated caries, as a non-conductor and astringent, but as it darkens the tooth, I gave it up.

I have also used hydronapthol crystals, heated to a resinous mass, and worked into fine canals, and covered with copper, with good results. It is especially applicable to teeth of the lower jaw.

In capping exposed pulps, where I am afraid of irritation, I use a saturated solution of hydronapthol or listerine as a germicide, applied when warm, and capped with paper or sheet lead, with a covering of cement, preferably oxysulphate, the latter not being so harsh as most of the calcined oxyphosphates.

Before closing the paper I will mention some of the popular antiseptics of the day. The virtues of carbolic acid, creosote and the essential oils are generally understood, and need no word here, with the exception, perhaps, of the oil of peppermint, which is of late attracting more than a passing notice.

The latest *International Medical Annual*, in speaking of the oil of peppermint, says it is readily diffusible, is superior to iodoform, in that it does not mask in any way the odor of any of the discharges. It possesses the special property of checking suppuration, independently of its antiseptic action; is as powerful as, but more penetrating and diffusible than, corrosive sublimate; is absolutely harmless, and is consequently the safest and best of all known antiseptics.

The peculiar action or influence of iodoform over the production of pus, is not as yet quite satisfactorily explained, and it is said to have no germicidal action. Its odor is quite enough to warrant dentists in adopting some substitute.

Creoline, the so-called latest of the best antiseptics known, a proprietary preparation of the coal tar series, resembles very much, and probably is the same thing as pixine, used in England for many years; is unirritating and a cheap disinfectant, but giving an unpleasant or shoppy odor to the office. It is slightly astringent.

Copper amalgam, I am inclined to believe, is an admirable antiseptic filling, and if judiciously used, is an important addition to any operating table.

By means of the rubber dam, an operation on the teeth may be more safely treated with good germicides.

The saliva ejector also plays its part in connection with cleanliness in an operation, and should always be used with patients who have difficulty in swallowing the saliva while the rubber dam is in place.

While it is a very difficult thing to attain absolute antisepticism, yet, practically, it may be gained by attention to details.

Thorough cleanliness is a preventive of sepsis.

Germicides, well applied, in sufficient quantities or strength, will inhibit the development of aseptic germs.—*Archives of Dentistry.*

